# Static and Dynamic Assessment of Intelligence in ADHD Subtypes

**DOI:** 10.3389/fpsyg.2022.846052

**Published:** 2022-02-25

**Authors:** Rosa Angela Fabio, Giulia Emma Towey, Tindara Caprì

**Affiliations:** ^1^Department of Clinical and Experimental Medicine, University of Messina, Messina, Italy; ^2^Department of Life and Health Sciences, Link Campus University, Via del Casale di S. Pio V, Rome, Italy; ^3^Institute for Biomedical Research and Innovation (IRIB), National Research Council of Italy (CNR), Messina, Italy

**Keywords:** intelligence, assessment, cognitive development, ADHD, learning

## Abstract

There is a debate about the measure of IQ in children with ADHD. Some studies report that, compared to static assessment procedures, dynamic assessment of intelligence can better measure cognitive modifiability and plasticity. The present study was designed to examine children belonging to different ADHD subtypes (inattentive, hyperactive/impulsive, and combined) in terms of both static (WISC scores) and dynamic measures (Modifiability index). Thirty-four children (12 ADHD-I, 10 ADHD-H, and 12 ADHD-C) were compared to a sample of 27 typically developing children. Results indicate that only the inattentive and the combined subtypes, compared with the normative sample, show lower IQ scores. The ADHD-I group presents generally low WISC scores and ADHD-H presents generally high WISC scores. Moreover, the ADHD-C group shows a low static score and a high dynamic score, indicating a wide breadth of Vygotskian children’s zone of proximal development. Static and dynamic measurements together can indeed be considered a comprehensive examination of intelligence levels in ADHD children and may be essential in predicting learning capacities.

## Introduction

Traditionally, the measures of intelligence are obtained by testing either a ratio of mental age to chronological age or a score of deviation from an expected test performance by age. Such measures are referred to as “static assessment procedures,” and one of the major characteristics is that they emphasize previously acquired knowledge in terms of intelligence or achievement scores; they do not incorporate modifications aimed at increasing levels of performance into their procedure (Feuerstein et al., 1979; Carlson and Mann, 2002; Fabio, 2005).

Dynamic measures of intelligence are generally obtained by administrating novel problem-solving tasks to the subjects, supplying them with gradual and balanced assistance that progressively discloses the solution of the problem, and determines the amount of aid the learner needs to be able to solve the problem. This aid is inversely proportional to the modifiability index. The modifiability index, that is the general propensity to change, can be considered a more appropriate measure of intelligence (Tzuriel and Kaufman, 1999; Fabio, 2005; Fabio and Antonietti, 2012; Fabio and Urso, 2014; Fabio et al., 2015; Fabio, 2017).

Evidence on the effectiveness of dynamic testing confirms that it has considerable value for predicting children’s learning potential. Both, age and static measures of intelligence are positively related to the breadth of Vygotskian children’s zone of proximal development as determined by dynamic testing (Vygotskij, 1978). The performance in dynamic measures is strongly age-dependent, in the sense that older children perform better than younger ones (Mous et al., 2017); moreover, static measures are related to dynamic measures meaning that higher levels of classic or static intelligence tests are associated with high levels of dynamic measures.

Fabio (2007a,b) conducted a study in which both static and dynamic standardized measures of intelligence were applied in 287 children (mean age = 8 years and 2 months). By combining both static and dynamic grouping levels, five different profiles emerged: children with low static measures and low and medium dynamic measures; children with medium static measures and medium and high dynamic measure; children with high static measures and high dynamic measures. Among these, two profiles needed to be focused: children with low static indexes and medium modifiability indexes (49%) and children with medium static indexes and high modifiability indexes (18%). The children belonging to these two profiles were those who perform poorly when left on their own and often perform substantially better when given appropriate instructional intervention. A post-hoc analysis of socio-demographic and clinical data showed that the 76% of these children fall in two categories: subjects with low socio-economic background and subjects with hyperactivity. For the first group, the rationale is that dynamic measures can add predictive information seeing as the tasks within such assessment are somewhat beyond the learner’s competence, in terms of pre-existing knowledge, and therefore not related to low sociocultural background. (Missiuna and Samuels, 1989; Tzuriel and Kaufman, 1999; Vialle, 1999; Fabio, 2005). For the second group the rationale is that, since static measures of intelligence reveal the product of learning and not the process, they may underestimate gifted students with Attention Deficit Hyperactivity Disorder (ADHD). Even though Intelligence Quotient of the ADHD is normally distributed, the frequent association with disruptive behaviors in gifted ADHD students, limit the possibility to observe the indicators of high ability when left on their own (Fabio and Caprì, 2015, 2017; Mohammadhasani et al., 2015, 2018; Sempio et al., 2016; Martino et al., 2017; Fabio et al., 2018, 2019; Caprì et al., 2020).

It is likely that ADHD has a uni-directional effect on intelligence in a number of ways (Goldstein, 2011). The impact of limited self-control and impaired sustained attention may, to a small degree, diminish the acquisition of intellectual skills. However, to a larger degree ADHD is likely to interfere with skill application and the efficient test taking strategies necessary to perform well on intelligence tests. Most of the data suggest, however, that less than 10% of the variance in Verbal IQ is due to ADHD. Thus, the population of individuals with ADHD generally falls within a normal distribution in terms of intellectual skills. One might expect that 3 to 5% of those with gifted intellect will meet the symptom criteria for ADHD. In the study previously cited, Kaplan et al. (2001) examined 63 children with ADHD, 69 children with reading difficulties (RD) and 68 children with comorbid symptoms (ADHD + RD) by administering WISC-III. Results indicated that the distributions of estimated full scale IQs for each of the three groups of children did not differ significantly. Most children in each group scored in the average range and ADHD children were no more likely to have an above-average IQ than were other children. Within the research that has analyzed intellectual performance on static measures, conducted a study on the performance of ADHD children on the WISC-III. The sample included 43 clinically diagnosed ADHD children between the ages of 7 and 13 years. The results revealed that nine of the subtest scores showed significant differences between ADHD children and the normative sample. In particular, the former scored higher on the picture completion, block design, and mazes subtests but significantly lower on the information, coding, picture arrangement, arithmetic, symbol search, and digit span subtests. Furthermore, the ADHD subjects scored significantly lower on the processing speed index and freedom from distractibility index scores, as well on the verbal IQ than the normative group.

Due to the controversy about the measure of IQ in children with ADHD, the present study was designed to examine children belonging in each of ADHD subtypes and typically developing students in terms of both static and dynamic measures. More specifically, the first question addressed in the study was whether a precise identification of ADHD subtypes could help us to better understand the role of each of these on static measures of intelligence, with reference to both quality and quantity of performance. The second question was to analyze if each subtype of ADHD shows different levels of dynamic measures, thus giving the possibility to predict children’s learning potential. Moreover, the answers to these questions should allow us to understand which subtype performs poorly when left alone (static measures) and better when given appropriate instructional intervention (dynamic measures), in other words, which ADHD subtype presents a wider breadth of Vygotskian children’s zone of proximal development as determined by dynamic testing.

## Materials and Methods

### Participants

The participants in this study were selected from a sample of 987 students attending public schools in Lombardy, a region of Northern Italy, and Sicily, a region of Southern Italy. Students ranged in age from 8 to 10 years and were attending either the 4th or 5th grade of primary school. A wide sample of schools had been involved in previous on-the-job training courses addressed to teachers, who were contacted and asked to collaborate in the investigation. The procedure described below was followed in all schools that decided to participate. Inclusion criteria for the ADHD group were: (1) a positive screening for ADHD based on the Deficit Attention Teacher Scale (DATS; Marzocchi and Cornoldi, 2000); (2) a negative screening for disruptive behavior disorder (DBD) based on the disruptive behavior disorder Rating Scale (DBDRS; Marzocchi et al., 2001); (3) a clinical diagnosis from a specialized psychologist; (4) no learning disabilities or neurological and psychiatric disorders.

#### ADHD Group

Eighteen items compose DATS, corresponding to the symptom domain of ADHD as described in the Diagnostic and Statistical manual of Mental Disorders (DSM-5; American Psychiatric Association, 2013). Two scores can be obtained: a measure of distractibility or inattention (I) and a measure of hyperactivity (H). Children can meet ADHD-I criteria (inattentive subgroup), ADHD-H criteria (hyperactive subgroups), or ADHD-C criteria (combined: inattentive+hyperactive subgroups). In this experiment, inclusion in the ADHD condition was based on cutoff scores in both subscales (I and H) and on a clinical assessment carried out by a specialized psychologist. The presence of other disorders was excluded by normal DBDRS scores and by the clinical interview. The ADHD group was composed of 12 students who met ADHD-C criteria, 12 who met ADHD-I criteria, and 10 who met ADHD-H criteria. No child had a history of brain damage, epilepsy, psychosis, or anxiety disorders.

#### Typical Developing Group

The sample of the initial 987 students who obtained DATS and DBDRS scores in the normal range, who were not included in any clinical group, and of children not diagnosed by the school psychologists with behavioral, emotional, and/or relational problems constituted the basis to form the TD group. Twenty-seven students were randomly extracted from such a sample. Their gender and age were considered in order to choose students who could constitute a group whose boy/girl ratio and whose mean age matched the clinical groups. Among TD children who were selected based on gender and age criteria, only children who also have DATS and DBDRS scores as 0 and had no clinical disorders, were included in the final TD group. The characteristics of ADHD and control children are summarized in Table 1.

**Table 1:** Characteristics of the participants.

Groups	Measures	Values
ADHD-I	*N* of boys/girls	10/2
	Age, *M* (SD)	8.9(1.4)
	SDAI-distractibility, *M* (DS)	18.2(2.45)
	SDAI-hyperactivity, *M* (DS)	4.1(6.01)
ADHD- C	*N* of boys/girls	10/2
	Age, M (SD)	8.8(1.2)
	SDAI-distractibility, *M* (DS)	17.2(3.45)
	SDAI-hyperactivity, *M* (DS)	18.1(6.01)
ADHD-H	*N* of boys/girls	8/2
	Age, *M* (SD)	9.01(1.25)
	SDAI-distractibility, *M* (DS)	4.2(2.45)
	SDAI-hyperactivity, *M* (DS)	17.1(6.01)
TD	*N* of boys/girls	20/7
	Age, *M* (SD)	9.03(2.01)
	SDAI-distractibility, *M* (DS)	3.29(0.2)
	SDAI-hyperactivity, *M* (DS)	1.2(0.32)

As expected, the TD group differed significantly on the items of distractibility [*F*(3, 60) = 21.60, *p* < 0.001] and on items of hyperactivity subscales [*F*(3, 60) = 18.11, *p* < 0.001]. The gender effect within the ADHD groups was not statistically significant.

### Instruments

The tests used in this work were the static and dynamic measures of intelligence.

#### Static Test: WISC

The WISC-III consists of 10 subtests and takes between 50 and 60 min to complete. The two subscales are Verbal IQ and Performance IQ. The child’s verbal IQ score is derived from scores on five of the subtests: information, vocabulary, arithmetic, comprehension, and similarities. The child’s performance IQ is derived from scores on the remaining five subtests: picture completion, picture arrangement, block design, object assembly, and symbol search.

#### Dynamic Test: DT

The items included in the dynamic testing were the result of a standardization work performed on a sample of 287 children aged from 7 to 10 (Fabio, 2003), which had met satisfactory criteria in statistic reliability. The psychometric evidence supporting Dynamic Test can be summarized as follows: Cronbach’s alpha = 0.97, *p* < 0.001, 1 month test–retest correlation = 0.71 (df = 234, *p* < 0.001). Internal reliability for the two subscales (learning and transfer) was, respectively, 0.88 and 0.86.

These dynamic testing items are problem-solving items that the child does not solve spontaneously, but in the course of the learning session. The test in its final issue contains 14 items, 7 of which are related to the learning phase, and another 7 to the transfer phase. The latter contain the same rules for problem-solving as the items related to the first phase plus a new rule that child must use to be able to find the solution. The items included for this age are as follows: (1) completion of a series of letters, (2) completion of a series of numbers, (3) completion of geometrical figures, (4) perceptive difference, (5) mental image superimposition, (6) chain of words, (7) simultaneous coordination of information.

**Figure 1:**
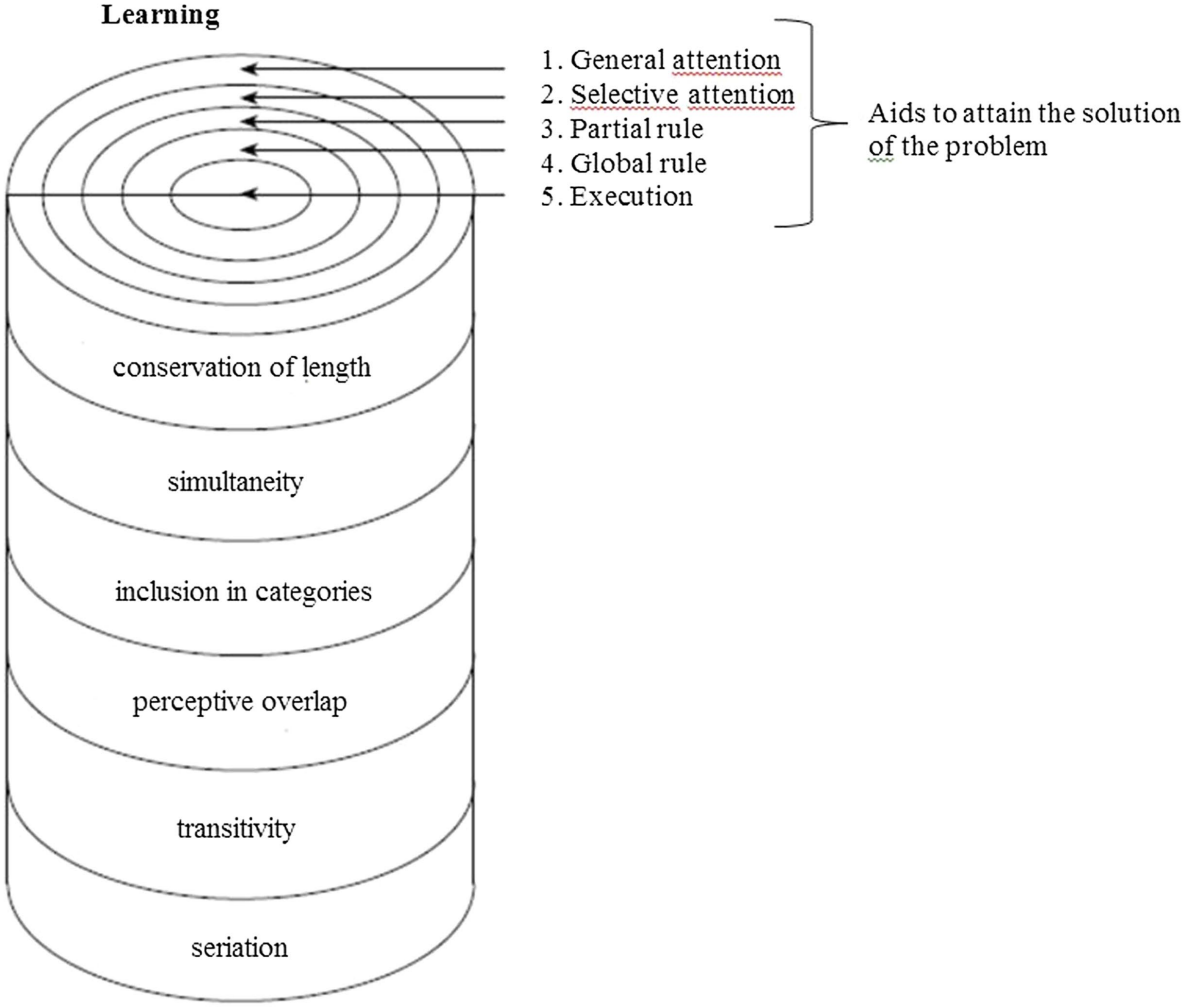
Learning phase.

Figure 1 shows the test structure: a three-dimensional cylindrical figure in whose sections are the 14 items of the two testing stages. The concentric circles represent the graded suggestions supplied to the child in order to achieve the solution of the problem. The stages identified for achieving the solution and constituting the progressive aid are 5. They go from the most “peripheral” (general attention) to the most “central” one (execution) where the child is taught operatively how to solve the problem.

### Procedure

The children were tested in a quiet area of the school in two sessions, each lasting approximately 1 h. In the different sessions, participants performed randomly the WISC and the Dynamic Test. The administration time of the WISC varied between 45 and 60 min. The test was entirely administered in one session. Each subtest was given separately.

With reference to the dynamic test, the experimenter showed the first problem to the child inviting him to solve it. The child was reminded that he could ask for help but he should try to perform the task with as little aid as possible. Aids were given one-by-one following a standard sequence. Once the correct solution of the first item was achieved, the same procedure was followed for all the other items: learning items first and then transfer ones. The mediator marked down how many aids had been requested by the child to achieve the correct solution and attributed relevant scores. Scores were established the other way around, that is, when the child solved the task with 1 aid only, he was assigned 5 points; with 2 aids 4 points; with 3 aids 3 points, with 4 aids 2 points, and with 5 aids 1 point. Summing up the scores in the learning stage we have the learning modifiability index (LMI), summing up the scores in the transfer stage we have the transfer modifiability index (TMI), the sum of the two indexes gives the general modifiability index (GMI: ranging from 16 to 80). Standard power calculations assuming a mix-model repeated-measure analysis plan were used to calculate sample size.

### Statistical Analysis

Data were analyzed using the Statistical Package for the Social Sciences (SPSS) version 20 (SPSS, Inc., Chicago, IL). The dependent variables (the results of each dynamic and static subtests and the overall scales) were analyzed through univariate analyses of variance entering Group (ADHD-C vs. ADHD- I vs. ADHD-H vs. Control) as the independent variable. The alpha-level was set to 0.05 for all statistical tests. In the case of significant effects, the effect size of the test was reported. The effect size was calculated using eta-squared *η*^2^ for ANOVA test. Cohen’s d effect size measure was applied for *t*-test. To correct for multiple comparisons, the Bonferroni correction was applied. The effects of gender within the ADHD groups were tested by using chi-square for independent sample. Statistical significance was determined at *p* < 0.05.

## Results

Table 2 reports means, standard deviations, and *F*-test for all groups and for each of the results of static test.

**Table 2:** Means, Standard deviations, and statistical test for each group and for each WISC subtest.

	ADHD-I		ADHD-C		ADHD-H		TD				
	*M*	SD	*M*	SD	*M*	SD	M	SD	*F*	*P*	D.F
Full scale WISC	89.4	9.68	98.6	8.65	114.6	18.59	103.44	17.59	6.22	0.001	3.59
Verbal IQ	74.70	10.31	98.3	6.68	113.5	18.76	98.37	20.48	8.87	0.001	3.59
Information	4.3	1.76	7.8	2.09	11.6	3.5	8.85	3.51	9.88	0.001	3.59
Similarities	6.9	5.13	12.3	2.26	15.8	2.69	12.03	4.66	7.81	0.001	3.59
Arithmetic	7.4	1.07	9.6	2.11	10.8	3.32	9.55	2.91	2.88	0.044	3.59
Vocabulary	4.6	3.34	8.4	4.45	12.1	6.24	8.44	7.1	3.32	0.026	3.59
Comprehension	7.02	4.26	10.7	2.83	10.7	2.98	9.88	3.66	2.21	0.097	3.59
Performance IQ	102.6	12.21	99.5	17.48	114.7	15.84	108.59	14.25	2.15	0.105	3.59
Picture completion	7.9	3.87	9	1.82	11.4	2.45	10	2.51	3.16	0.052	3.59
Picture arrangement	11.8	2.52	11.7	5.16	13.6	4.69	11	3.43	1.08	0.362	3.59
Block design	11.7	3.05	10.1	4.14	12.8	3.96	12.03	4.01	0.89	0.45	3.59
Object assembly	11.7	2.9	10.3	1.05	12.8	2.61	11.81	2.61	1.73	0.17	3.59
Symbol search	8.9	2.42	8.7	3.4	10.1	2.6	11.48	2.76	3.53	0.021	3.59

With reference to the full scale WISC IQ, the one-way ANOVA indicated that there is a main effect of Group [*F*(3, 59) = 6.22, *p* < 0.01, *η*^2^ = 0.12]. Follow-up *t*-tests revealed that children with ADHD-H presented higher IQ scores than children with ADHD-I (*t* = 29.2, *p* < 0.01, *d* = 0.65), children with ADHD-C (*t* = 17.31, *p* = 0.0, *d* = 0.54) and normally developing children (*t* = 12.1, *p* = 0.038, *d* = 0.22). Children with ADHD-I showed lower IQ scores than children with ADHD-H (*t* = 29.2, *p* < 0.01, *d* = 0.61) and TD children (*t* = 17.04, *p* = 0.015, *d* = 0.35).

With reference to information, the one-way ANOVA indicated that Group show significant statistical effect [*F*(3, 59) = 9.88, *p* < 0.01, *η*^2^ = 0.14]. Follow-up *t*-tests revealed that children with ADHD-I presented lower information scores than children with ADHD-H (*t* = 6.5, *p* < 0.01, *d* = 0.33), children with ADHD-C (*t* = 7.30, *p* < 0.01, *d* = 0.44) and normally developing children (*t* = 4.55, *p* < 0.01, *d* = 0.37).

With reference to vocabulary, the one-way ANOVA indicated that Group show significant statistical effect, *F*(3, 59) = 3.32, *p* < 0.02, *η*^2^ = 0.09. Follow-up *t*-tests revealed that children with ADHD-I presented lower vocabulary scores than children with ADHD-H (*t* = 8.97, *p* < 0.01, *d* = 0.45), children with ADHD-C (*t* = 3.13, *p* < 0.02, *d* = 0.23) and TD children (*t* = 3.40, *p* < 0.01, *d* = 0.35). In this case ADHD-C, ADHD-H, and TD children did not differ.

With reference to similarities, the one-way ANOVA indicated that Group show significant statistical effect, *F*(3, 59) = 7.81, *p* < 0.01, *η*^2^ = 0.23. Follow-up *t*-tests revealed that children with ADHD-I presented lower similarities scores than children with ADHD-H (*t* = 8.90, *p* < 0.01, *d* = 0.55), children with ADHD-C (*t* = 5.13, *p* < 0.01, *d* = 0.43) and TD children (*t* = 5.40, *p* < 0.01, *d* = 0.44). Children with ADHD-H presented higher similarities scores than children with ADHD-I (*t* = 12.5, *p* < 0.01, *d* = 0.65), children with ADHD-C (*t* = 3.3, *p* < 0.01, *d* = 0.33) and TD children (*t* = 3.55, *p* < 0.01, *d* = 0.35). In this case ADHD-C and TD children did not differ.

With reference to symbol search, the one-way ANOVA indicated that Group show significant statistical effect, *F*(3, 59) = 3.53, *p* < 0.02, *η*^2^ = 0.09. Follow-up *t*-tests revealed that children with ADHD-I presented lower symbol search scores than TD children (*t* = 2.58, *p* < 0.01, *d* = 0.27). Furthermore children with ADHD-C presented lower symbol search scores than TD children (*t* = 2.78, *p* < 0.01, *d* = 0.26). In this case ADHD-H and TD children did not differ.

Table 3 reports means, standard deviations, and test F for all groups with reference to the learning phase, the transfer phase, and the full scale.

**Table 3:** Means, Standard deviations, and statistical test for each group and for each subtest of the DT.

	ADHD-I		ADHD-C		ADHD-H		TD				
	*M*	SD	*M*	SD	*M*	SD	*M*	SD	*F*	*P*	D.F
Learning phase DT	21.2	1.87	20.5	2.59	23.9	4.86	22.11	3.95	1.62	0.195	3.59
Transfert phase DT	22	4.02	23.5	3.97	28.8	5.28	26.59	4.5	5	0.004	3.59
Full scale Dynamic Test	43.2	4.61	44	6.21	52.7	9.69	48.7	7.88	3.62	0.019	3.59

With reference to the full scale of dynamic testing, the one-way ANOVA indicated that Group show significant statistical effect both with reference to the full scale (*F*(3, 59) = 3.62, *p* < 0.01, *η*^2^ = 0.12) and with reference to the test of the transfer phase, *F*(3, 59) = 5.0, *p* < 0.01; with reference to transfer phase, follow-up *t*-tests revealed that children with ADHD-I presented lower dynamic testing scores than children with ADHD-H (*t* = 6.80, *p* < 0.01, *d* = 0.45), and TD children (*t* = 4.59, *p* = 0.038, *d* = 0.24). Children with ADHD-C showed lower IQ scores than children with ADHD-H (*t* = 5.30, *p* < 0.01, *d* = 0.45).

With reference to the full scale of dynamic testing, children with ADHD-H showed higher dynamic testing scores than children with ADHD-I (*t* = 9.50, *p* < 0.01, *d* = 0.58), and children with ADHD-C (*t* = 3.36, *p* < 0.01, *d* = 0.33).

The third question addressed in this study was to examine the dynamic measures of intelligence in each of the ADHD subtypes to better understand which subtype performs poorly when left alone (static measures) and better when given appropriate instructional intervention (dynamic measures), in other words, which ADHD subtype present a wider breadth of Vygotskian children’s zone of proximal development as determined by dynamic testing. In the present study, the classification of participants was based on standardization measures of previous works (Fabio, 2005) in which the children who had achieved the worst scores in static indexes (13%) and those who had the best scores (13%) were taken into account for the definition of the categories relevant to static testing. In the same way, the children, who achieved the worst scores in the present work, were defined as children with “low static measures.” Those who achieved the average scores were defined as children with “medium static measures.” Finally, those with the best scores were defined as children with “high static measures.” In the same way, using dynamic measures, we make reference to the children who had achieved the worst scores (13%) and those with the best scores (13%) in the standardized modifiability indexes and were defined children with low and high modifiability. Table 4 shows the distribution of each subject in each subtype with reference to the relationship between static and dynamic measures.

**Table 4:** Frequency of distribution of the Groups in relation to static and dynamic measures.

	ADHD-I	ADHD-C	ADHD-H	Control
**Static/dynamic measures**
low-low	9	1	_	2
Low-medium	3	4	_	5
medium-medium	_	3	1	13
medium-high	_	4	2	2
high-high	_	_	7	5
Total	12	12	10	27

*Chi-square (Yates corrected) = 5.23, p < 0.05.*

**Figure 2:**
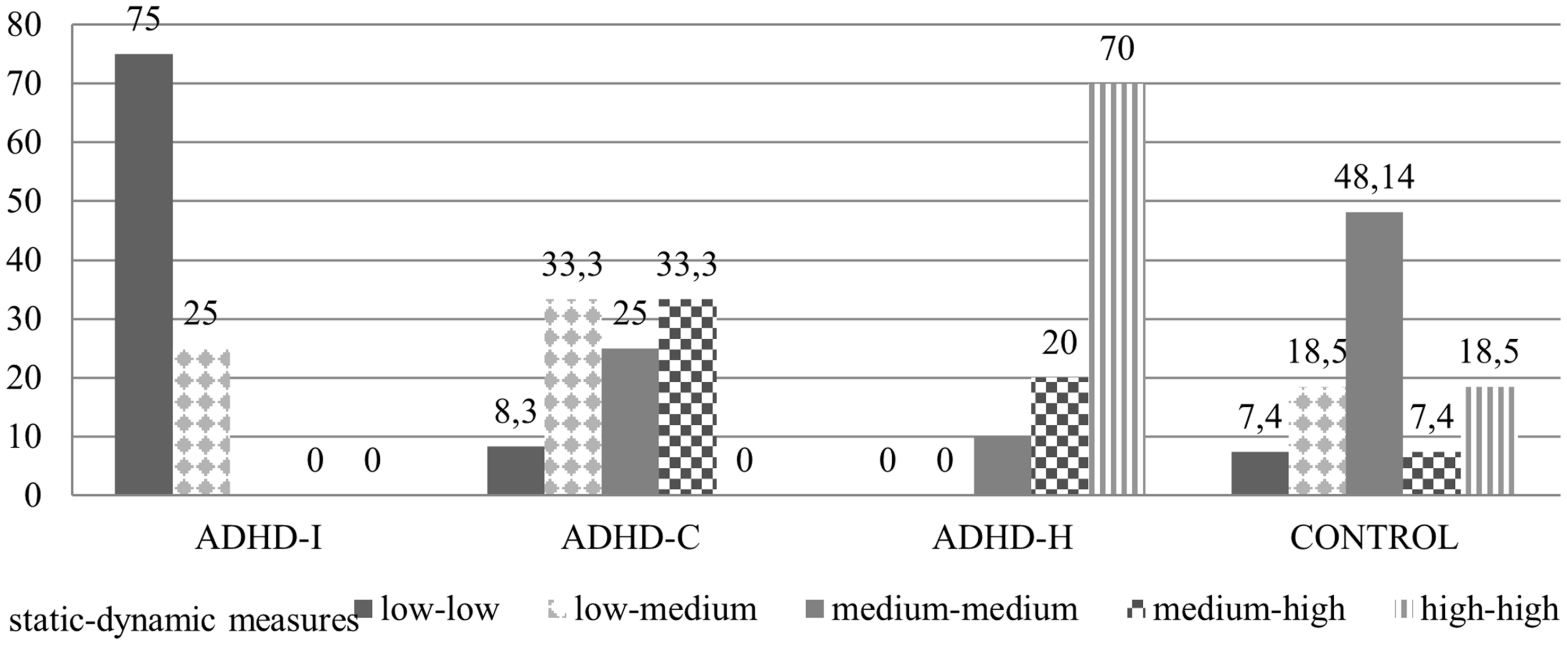
Response rates.

Figure 2 shows the percentage of participants that presents different combinations of static and dynamic measures.

## Discussion

The present study indicated that only the ADHD-I and ADHD-C groups, when compared with the TD group, show lower scores in comprehension, picture completion, picture arrangement, block design, and object assembly. By considering separately ADHD subtypes, more information emerged clarifying some discrepancies between the different authors in the field of static measures. Some studies that adopted mixed groups, generally show that students with ADHD have slightly lower IQs than control samples. The distinction into different subtypes helps us to illustrate that this can be applied only to ADHD-I which presents general low WISC scores as opposed to ADHD-H which presents general high WISC scores. Other studies indicated that the distribution of estimated Full Scale IQs (FSIQ) for each of the three groups of children did not differ significantly from a normal distribution, with most children (more than 50%) in each group scoring in the average range (Kaplan et al., 2001). These results in the present study can be applied mainly to ADHD-C, seen as it is the group that presents a normal distribution. In this subtype, the range of IQ is from severely intellectually disabled to the gifted range. Traditional IQ tests may underestimate intelligence in these children because (a) IQ subtests that assess mental math and digit span also require working memory, and (b) inattention can lower IQ from 2 to 5 Full Scale IQ points (Jepsen et al., 2009). For this reason, dynamic measures of intelligence in this work give us an important direction. The ADHD-C are children who perform poorly on static measures and respond quite differently to instruction suggesting that dynamic measures can provide information over and above that available from static tests. Dynamic testing reduces the possibility that a child who can profit from instruction mediation is denied opportunities to learn because of a poor score on a static assessment.

However, the results of the present study should be evaluated with caution, because the sample size is small. Thus, future research should examine dynamic and static tests using a larger sample size to ensure adequate power. Despite this limitation, this study is an indication of the importance of a wider evaluation of cognitive performance in predicting learning capacities in ADHD children.

Moreover, static and dynamic test assessment of cognitive ability has been also investigated in other domains, such as autism spectrum disorder (ASD). As regards to this, some studies have examined different methods of modeling or investigating cognitive ability among individuals with ASD, providing insights into the cognition-based neural dynamics of subjects with ASD. For example, a recent study has indicated that coupling strength can be a potential biomarker to identify cognitive status at a higher discrimination rate in ASD (Wadhera and Kakkar, 2020a). Another study has demonstrated that the brain network of subjects with ASD has become more segregated and less integrated when they performed a task with cognitive load, reflecting more involvement of intra-regions over inter-regions (Tanu and Kakkar, 2020; Wadhera and Kakkar, 2020b). These recent studies show the correlation between complex graph skills, cognitive ability, and learning, and, they further suggest that neural metrics can predict the behavioral performance of the individuals in visual-motor tasks. Hence, static and dynamic measures of cognitive ability can be studied in different domains, producing insights into the intellectual functioning.

## Conclusion

Two main conclusions emerge from the present study, the first is that static and dynamic measures together can give us more information about the intelligence of children with ADHD. The second is that although the ADHD occurs in three different clinical features (the three ADHD subtypes), it is necessary to reconsider the inclusion of ADHD-H subtype in a different framework in which behavioral but not cognitive problems are considered, given that only the ADHD-I and ADHD-C groups have shown a poor performance in comprehension, picture completion, picture arrangement, block design, and object assembly.

## Data Availability Statement

The raw data supporting the conclusions of this article will be made available by the authors, without undue reservation.

## Ethics Statement

The studies involving human participants were reviewed and approved by University of Messina. Written informed consent to participate in this study was provided by the participants’ legal guardian/next of kin.

## Author Contributions

RF conceived and designed the analysis and performed the analysis. TC and GT collected the data and contributed data and analysis tools. GT wrote the paper. All authors approved the manuscript.

## Conflict of Interest

The authors declare that the research was conducted in the absence of any commercial or financial relationships that could be construed as a potential conflict of interest.

## Publisher’s Note

All claims expressed in this article are solely those of the authors and do not necessarily represent those of their affiliated organizations, or those of the publisher, the editors and the reviewers. Any product that may be evaluated in this article, or claim that may be made by its manufacturer, is not guaranteed or endorsed by the publisher.
